# Shear Wave Elastography in the Differentiation of Nonfibrotic Versus Fibrotic Liver Disease in Children: A Prospective Study With Histological Correlation

**DOI:** 10.1097/PG9.0000000000000156

**Published:** 2021-12-10

**Authors:** Hanna Hebelka, Charlotte de Lange, Håkan Boström, Nils Ekvall, Kerstin Lagerstrand

**Affiliations:** From the * Department of Pediatric Radiology, Sahlgrenska University Hospital, Gothenburg, Sweden; † Institution of Clinical Sciences, Sahlgrenska Academy, University of Gothenburg, Gothenburg, Sweden; ‡ Department of Pediatric Medicine, Sahlgrenska University Hospital, Gothenburg, Sweden; § Department of Medical Physics and Techniques, Sahlgrenska University Hospital, Gothenburg, Sweden.

**Keywords:** classification, ultrasonography, elastography imaging techniques, liver, fibrosis, pediatric

## Abstract

To evaluate the diagnostic accuracy of ultrasound shear wave elastography (SWE) prospectively and to determine cutoff value for nonfibrotic liver tissue in children with suspected or established liver disease.

**Methods:**

In 90 consecutive pediatric patients, standardized 2D-SWE was performed during general anesthesia and free breathing. Liver stiffness was estimated with SWE followed by a percutaneous biopsy from the corresponding area. SWE values were compared with histology with fibrosis scored according to Batts & Ludwig classification (grade 0–4 = F0–F4) and to hepatic biomarkers.

**Results:**

Four patients with SWE interquartile range (IQR)/median ≥ 30% kPa were excluded. The remaining 86 children (59% males) had a mean age = 10.2 years (0.1–18). The distribution of individuals with median (min;max) SWE values (kPa) within each fibrosis grade were; F0[n = 10; 5.0(3.4;6.3)], F1[n = 24; 5.0(3.6;8.7)], F2[n = 32; 5.8(3.5;13.4)], F3[n = 12, 7.5(4.0;14.4)], and F4[n = 8; 12.5(6.6;21.0)]. There was a significant difference between fibrosis grades (0.03 > *P* < 0.002) except between F0 and F1 respectively between F1 and F2. The AuROC differentiating F0-1 from F2-4 was 0.77(95% CI: 0.67-0.87). A cutoff SWE value of ≤4.5 kPa yielded 90% sensitivity and 68% specificity to rule out significant fibrosis (F2–F4). Out of the 18 children (21%) with SWE value ≤4.5 kPa, 12 had grade F0-1 and 6 had F2, although including some confounders for increased SWE measurements as steatosis/hepatitis/cholestasis.

**Conclusions:**

2D-SWE ultrasound can reliably distinguish no/mild (F0/F1) from moderate/severe (F2–F4) fibrosis in children with suspected/established liver disease with good sensitivity and acceptable specificity. Our results show that in pediatric patients, when the indication for biopsy is to rule out significant fibrosis, SWE can be considered an alternative.

## Abstract 2

Supplemental Digital Content is available in the text.

**What Is Known**

- Ultrasound shear wave elastography (SWE) is an evolving tool to estimate liver fibrosis noninvasively.
- The diagnostic performance of pediatric SWE needs further systematic validation before considering an implementation in pediatric clinical routine.

**What Is New**

- Prospective evaluation, using concurrently acquired SWE measures and biopsy, revealed SWE able to distinguish no/mild from moderate/severe fibrosis in children with suspected/established liver disease with good sensitivity and acceptable specificity.
- SWE measures had a moderate correlation with histologic fibrosis grade but neither with inflammation nor hepatic serological markers.
- Findings suggest that SWE, also with free breathing, can be considered an alternative to biopsy in pediatric patients when the indication for biopsy is to rule out significant fibrosis.

## INTRODUCTION

Chronic liver disease is a significant and increasing health problem in children, associated with progressive fibrosis and cirrhosis (1,2). The leading causes for chronic liver disease in children include nonalcoholic fatty liver disease (NAFLD), hepatitis (infectious, autoimmune, or drug-related), genetic diseases (a-1 antitrypsin deficiency, cystic fibrosis, biliary atresia, etc.), and congestive hepatopathies. It is of paramount importance to detect and stage liver fibrosis for adequate treatment, surveillance, and prognosis. Today, biopsy of the liver is gold standard to obtain detailed tissue characteristics regarding if, and to what extent, the liver is affected by fibrosis. However, liver biopsies are limited by its invasive nature, associated complications and potential sampling errors (3). In addition, within the pediatric population, biopsies require general anesthesia.

During the last decade, noninvasive methods to evaluate liver fibrosis, such as ultrasound (US) elastography and magnetic resonance (MR) elastography, have been proposed to quantify liver stiffness (LS) as a measure of fibrosis (2,4–8). Within the pediatric population, in which MR elastography is more time consuming and often requires anesthesia (5), US shear wave elastography (SWE) is increasingly used to diagnose and estimate liver fibrosis in children and adolescents (2,4,6,9). Traditionally elastography has been performed by nonimaging techniques that use an external mechanical push, so called vibration controlled transient elastography techniques. A drawback with these nonimaging techniques is the lack of control for exactly where the measurement is obtained as for the reliability and quality of the measurement. Newer techniques provide information of the LS by measuring the speed of shear waves, emitted by an acoustic radiation force impulse (ARFI) using a B-mode imaging over the liver, providing measurement quality parameters (6). Recently, the ARFI technique 2D-SWE was reported to correlate moderately with fibrosis grade of severity in a pediatric study (5).

Prospective SWE studies in large pediatric cohorts, comparing liver biopsy with simultaneously obtained SWE data are warranted to evaluate the diagnostic performance of SWE and how this technique can be implemented in the clinical evaluation and treatment decision of pediatric patients (6,10). A recent retrospective study reported that 2D-SWE can be used to distinguish between no/mild fibrosis and moderate/sever fibrosis in the pediatric population with good specificity and sensitivity (11). A few other pediatric studies have evaluated the relationship between SWE and liver biopsies; however, majority are limited in study design being either retrospective, small in sample size, not including infants or performed with a large interval between biopsy and SWE (5,7,8,11–19). For example, despite the fact that the study by Farmakis et al. was prospective and included 70 children, a different ultrasound system was used with a time interval up to 30 days between SWE measurements and biopsy allowed and SWE sampled either in awake state or under general anesthesia, factors that might impact the results (5).

It was hypothesized that the need for biopsies in children with liver disease can be reduced by establishing US SWE measures that differentiate clinically insignificant grades of liver fibrosis to more severe grades and cirrhosis. This prospective study therefore aimed to evaluate the diagnostic accuracy of 2D-SWE and to determine cutoff value for nonfibrotic tissue in children with suspected or established liver disease, to possibly avoid unnecessary biopsies.

## METHODS

### Patients

Between March 2019 and October 2020, all patients scheduled for a clinically indicated liver biopsy by the medical department at the Queen Silvia Children’s Hospital, Gothenburg, Sweden were consecutively invited to participate. Inclusion criteria were any form of suspected or confirmed liver disease, age between 0 and 18 years and consent by the child/parents/guardians to participate in the study. When inadequate language-skills constituted an obstacle to sufficiently understand written and oral information, patients were not invited to participate. Ninety patients were enrolled. This prospective study was conducted according to the Declaration of Helsinki. Ethical approval (Dnr 634-18) was given by the regional ethics review board, and oral and written informed consent was obtained from all participants and/or their parents/guardians.

**FIGURE 1.**
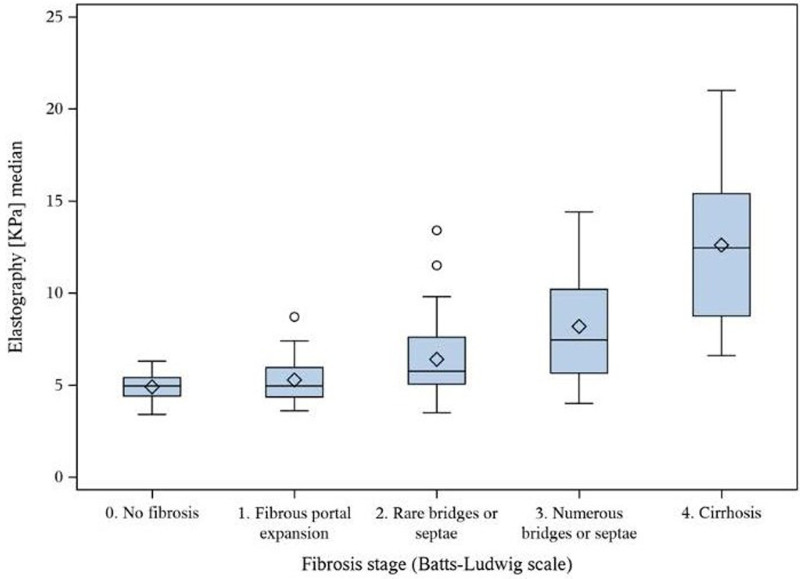
Box and whisker plot of 2D-SWE values for each fibrosis grade F0-4.

SWE-measurements During anesthesia (fasting > 4 h) and free breathing, 2D-SWE (Canon Medical, Aplio i700) measurements were performed in the right liver lobe with an intercostal approach, applying minimal transducer (i8CX1) pressure. LS measurements, estimated with SWE (kPa), were performed according to the latest liver elastography consensus statement by the Society of Radiologist in Ultrasound (9). The patient was examined in a supine position with the right arm raised over the head. Whenever an intercostal sampling was not possible, as in partial liver transplants, a subcostal acquisition was performed in a midline position. LS was measured 1.5–3 cm below the liver capsule using continuous mode, where the median of 10 registrations with interquartile range (IQR)/median < 30% kPa was recorded (9). During the same anesthesia, following the SWE measurement, 1–2 percutaneous 18 Gauge biopsies were obtained from the corresponding area of the liver. SWE sampling was performed by one of five physicians specialized within pediatric radiology, trained in SWE.

### Histopathology Scoring

#### Grading of Fibrosis Severity

Fibrosis severity was scored according to the Batts & Ludwig classification (stage 0–4 = F0–F4) (20), that is, the scoring system clinically used at the study site to evaluate all liver biopsies. Blinded to the SWE-measurements, one board-certified pediatric pathologist with 6 years of experience, conducted the scoring of all biopsies. If the biopsy included more than one fibrosis grade, the highest grade was registered, even if only a minor part of the specimen included the higher score.

#### Inflammation Evaluation

Inflammation cannot be reliably determined with SWE and is considered as a confounding factor when assessing fibrosis using SWE (2,9,15,21). Inflammation in a specimen was categorized by an in-house descriptive scoring system, used clinically at our department for unspecified inflammation, as; none, mild, moderate, and severe. This score was used both when unspecified inflammation was present in the specimen and for conversion from scoring systems for specific types of inflammatory conditions such as chronic hepatitis and steatohepatitis.

The Batts & Ludwig classification (grade 0–4) (20) was used for specimens with chronic hepatitis and converted to the in-house scoring system accordingly; grade 0–1 (none), specimen including grade 2 (mild), grade 3 (moderate), and grade 4 (severe). The NAFLD score (0–3) was used in patients with steatohepatitis and converted to the in-house scoring system accordingly; grade 0 (none), grade 1 (mild), grade 2 (moderate), and grade 3 (severe). If biopsy scoring included more than one grade of inflammation, the highest grade was registered in the study protocol, even if only a minor part of the specimen included this higher score.

#### Steatosis Evaluation

Regarding steatosis, the histopathological specimens were evaluated in percent liver cells affected and graded according to the NAFLD score; <5% (score 0), 5–33% (score 1), 33–67% (score 2), and >67% score 3.

#### Comparisons to Biomarkers

The estimated SWE values were compared with both histology and hepatic serological markers age and BMI (Table 1). With few exceptions, all serological markers were collected the same day as the biopsy or 1–2 days before (mean 2 days, range 0–12 days).

**TABLE 1.** Correlation between SWE values and serological markers

				Spearman correlation coefficient ρ
Variable	n	Mean	SD	SWE value (kPa)
Age, y	86	10.2	5.6	(–)0.07
BMI (kg/m^2^)	73	19.3	4.8	(–)0.12
INR for protrobine time	82	1.1	0.24	0.26
AST (µkat/L)	75	1.7	3.26	(–)0.16
ALT (µkat/L)	84	2.0	3.67	(–)0.07
White cell count [×10*9/L]	73	8.2	14.2	0.07
Thrombocytes [×10*9/L]	85	260.8	122.6	(–)0.15
Gamma-GT [µkat/L]	79	1.2	1.83	0.19
Fibrosis	86			0.55
Inflammation	85			0.17

ALT = alanine aminotransferase; AST = aspartate transaminase; BMI = body mass index; INR = International normalized ratio; SWE = shear wave elastography.

#### Blinding

Physicians performing the index test were blinded to histopathological results, and the pathologist was blinded to elastography results.

#### Reliability Valuation

Interobserver reliability regarding the SWE measurement was performed in 27 individuals by 2 readers blinded to each other’s results. Immediately after SWE sampling by the first reader (10 repeated measurements generating one median value), a second radiologist performed likewise 10 measurements at the same position in the liver. It was considered impossible to perform intraobserver variation in a blinded fashion during the same anesthesia procedure and unethical to repeat during anesthesia at another time. To evaluate intraobserver reliability measurements on previously obtained multimode cine-loops SWE sampling, 10 repeated readings were performed in 20 individuals, blinded to the previous results, after approximately 2 months.

### Statistics

Descriptive statistics was used with n (%) presented for categorical variables and median (min;max) presented for continuous variables. To test for association trend between SWE values and grade of fibrosis respectively between SWE values and inflammation, the Jonckheere-Terpstra test was used. For pairwise comparison between groups of continuous variables, Fisher’s nonparametric permutation test was used. For comparison between groups of dichotomous variables, Fisher’s Exact test was used. Spearman rank correlation (rho) was used to assess the relationship between SWE measures and continuous variables.

Logistic regression analysis was performed for each independent variable to predict the outcome. Area under ROC-curve (AUC statistics) was calculated for description of goodness of predictors. Intraclass correlation coefficient (ICC) (Shrout-Fleiss reliability:single score) was used to evaluate observer variation. The data were analyzed using version 9 of the SAS System (version 9.4). A *P* < 0.05 was considered significant.

## RESULTS

### Patients Characteristics

During the study period, SWE and liver biopsy at the same occasion was performed in 90 patients. Four patients with SWE measurements of limited quality, that is, IQR/median > 30% kPa were excluded (2 split transplants, 1 cholestasis, 1 hepatitis). Two of the included individuals were examined twice, as they were referred for a second biopsy during the study period. Thus, in total, 84 unique individuals with 86 SWE and liver biopsies were included in the study. The mean age (SD) was 10.2 (5.6) years (range: 0.1–18 years) with 60% males. Nineteen percent of the children were 0–2 years of age. Indications for liver biopsy are presented in Table 2. Distribution of grade of fibrosis, inflammation, and steatosis within the cohort is displayed in Table 3.

**TABLE 2.** Indication for liver biopsy

**Indication for biopsy/clinical diagnosis**	**Number of individuals (biopsies**)
Portal hypertension	3
Autoimmune hepatitis	11 (12)
Livertransplants (of which 3 acute biopsies; 4 biopsies displayed some kind of rejection)	24 (25)
Unspecified—increased serological livermarkers	14
Cystic fibrosis	3
Primary and autoimmune sclerosing cholangitis	9
NASH	2
Hepatitis B	5
Unspecified cholestatic disease	4
IFALD	1
Neonatal cholestasis	1
Congenital disorder of glycosylation	1
Wilsons disease	3
Heart transplant with liver disease	1
Nieman pick type 1	1
alfa 1 antitrypsin deficency	1
IFALD = intestinal failure-associated liver disease; NASH = non alcoholic steatohepatitis.	

**TABLE 3.** SWE values for each grade of fibrosis F0-4 and distribution of histopathologic grading

**n = 86**		**Entire study cohort (n = 86**)		**Excluding inflammation and steatosis (n = 61**)			**Distribution of histopathologic grading inflammation and steatosis**			
**Fibrosis stage (Batts&Ludwig**)	**n (%**)	**Median SWE(kPa**)	**Min:max**	**Median SWE(kPa**)	**Min:max**	**Individuals (n**)	**Inflammation**	**n (%**)	**Steatosis**	**n (%**)
F0	10 (11.6%)	5.0	3.4; 6.3	4.9	3.4; 6.3	9	None	15 (17.6%)	0	76 (88%)
F1	24 (27.9%)	5.0	3.6; 8.7	4.9	3.6; 8.7	20	Mild	55 (64.7%)	1	7 (8%)
F2	32 (37.2%)	5.8	3.5; 13.4	5.7	3.5; 11.5	20	Moderate	12 (14.1%)	2	2 (2.3%)
F3	12 (14.0%)	7.5	4.0; 14.4	8.0	4; 14.4	8	Severe	3 (3.5%)	3	1 (1.2%)
F4 (Cirrhosis)	8 (9.3%)	12.5	6.6; 21.0	13.8	6.6; 16.7	4	Missing	1		

SWE = shear wave elastography.

### SWE Measurements and Histopathology

#### Grade of Fibrosis

**FIGURE 2.**
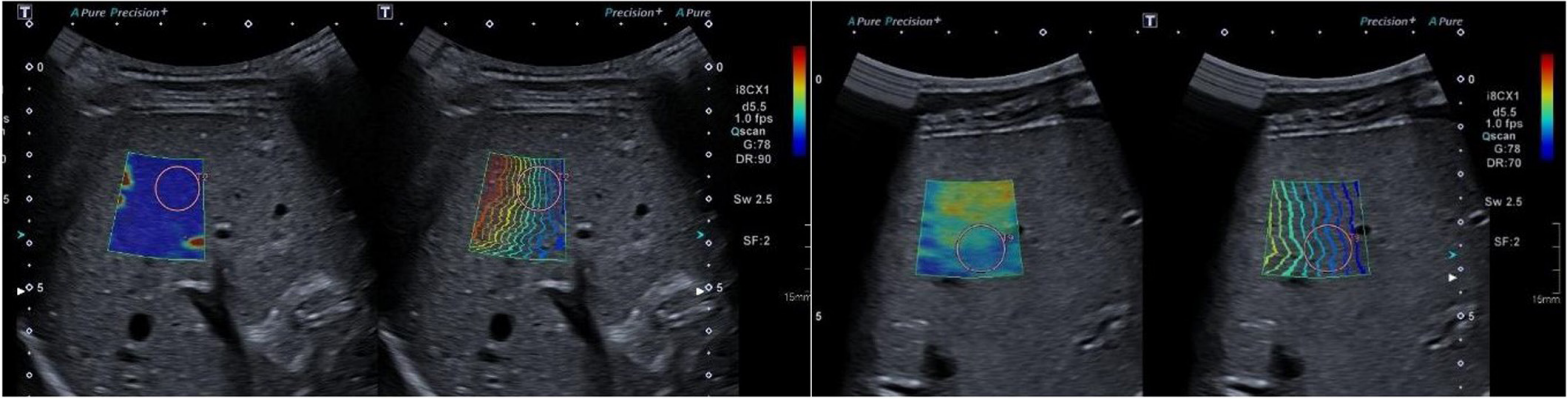
Example of SWE measurement in a normal liver (F0) (left) compared with measurement in a fibrotic liver (F3) (right). In the fibrotic liver an inhomogeneous color-map and increased distance between the shear waves is seen (right), reflecting increased liver stiffness displayed as a high SWE value (13.3 kPa). The SWE appearance in the normal liver to the left, displayed as 4.0 kPa.

Median liver SWE values within each grade of fibrosis are displayed in Table 3. Overall, median SWE values were significantly different between different grades of fibrosis (*P* < 0.0001) (Figure 1). The difference was significant for all (0.03 > *P* < 0.002) except between F0 and F1, respectively, F1 and F2. Example of SWE measurement in a nonfibrotic versus a fibrotic liver is displayed in Figure 2. There was a moderate correlation between SWE values and grade of fibrosis (*P* < 0.0001; ρ = 0.55).

#### Grade of Inflammation and Steatosis

The SWE values did not correlate with inflammation (*P* = 0.12; ρ = 0.17), and there was no significant difference in overall SWE measures between the different grades of inflammation (*P* = 0.099) (Table 1). No tests between individual grades were performed. Three patients had severe inflammation in their biopsies of which two individuals had F4 (10.2, respectively, 11.0 kPa) and one individual had F2 (7.2 kPa). To eliminate inflammation and steatosis as confounders, SWE values for each grade of fibrosis, excluding inflammation > grade 1 and steatosis > 1, are also displayed (Table 3).

The AuROC for differentiating F0-F1 from F2-F4 was 0.77 (95% CI: 0.67-0.87) (Supplemental Digital Content Figure 1, http://links.lww.com/PG9/A65, displaying the AuROC for differentiating F0–F1 from F2–F4). A cutoff SWE value of ≤4.5 kPa yielded 90% sensitivity and 68% specificity to rule out F2–F4. This corresponds to a value of approximately ≤1.24 m/s (yielding a 90% sensitivity and 71% specificity). Of the 18 children (21% of the cohort) with SWE value ≤4.5 kPa, 12 individuals had grade F0-1 whereas 6 had F2 (steatosis n = 2, AIH n = 1, hepatitis B n = 1, transplants n = 2, of which 1 had chronic cholestasis).

#### Correlation to Biomarkers

No correlation was found between SWE values and hepatic serological markers. Neither did SWE values correlate to age nor BMI (Table 1).

#### Reliability Valuation

ICC was excellent for both interobserver SWE measurement (ICC 0.96, 95% CI: 0.92-0.98/CV 0.11, 95% CI: 0.08-0.15) as well as for intraobserver measurements on repeated readings (ICC 0.97, 95% CI: 0.92-0.99/CV 0.08, 95% CI: 0.05-0.11).

## DISCUSSION

In this prospective study, free-breathing 2D SWE was performed under general anesthesia preceding liver biopsy assessed from the corresponding area of the LS measurement. SWE was shown to distinguish no/mild fibrosis from moderate/severe fibrosis in children with suspected or established liver disease with good sensitivity and specificity. A relatively large pediatric cohort, including many infants, a wide spectrum of diseases in addition to a study design ensuring good observer reliability, are all elements that strengthen the results of the current study.

US SWE, in especially the liver, have been increasingly studied in children the last decade (6,9,19,22). The research question we sought to answer was if a 2D-SWE cutoff value could be used in individuals with suspected liver disease, to rule out patients in whom significant liver fibrosis is unlikely and unnecessary biopsies potentially could be avoided. Current study confirmed that SWE can differentiate between nonfibrotic and moderate/severely fibrotic tissue with good performance in pediatric patients (2,5,8,11–14,19). This confirmation is important given that we address several limitations in previous studies, such as retrospective study design, multiple probes used, and biopsies not obtained at the same occasion as the SWE measurement (7,8,12–18). Since it is preferred to identify patients at risk for fibrosis, compared with erroneously exclude patients with fibrosis, a low cutoff SWE value was chosen (9). For the same reason, a high sensitivity was considered important while an intermediately high specificity was considered acceptable. With a cutoff SWE value of ≤4.5 kPa, the sensitivity and specificity to rule out F2–F4 was 90%, respectively, 68%. Of the 18 children (21%) with SWE value ≤4.5 kPa in the current cohort, 12 individuals had grade F0-1. In the 6 individuals with SWE value of ≤4.5 kPa and higher grade of fibrosis, none was graded higher than F2, which likely is overestimated (see later), and all had confounding factors like inflammation, cholestasis, or steatosis. Defining fibrosis is of course not the only reason to perform a liver biopsy but when the indication for biopsy is to rule out significant fibrosis in pediatric patients, biopsy could be omitted, or at least be reconsidered, in majority of individuals with SWE value of ≤4.5 kPa.

Other studies, differentiating no and mild fibrosis from moderate and severe fibrosis, have reported AuROC with sensitivity and specificity within the same range as in the current study (13,14,19). Alhashmi et al recently reported a AuROC of 0.75 for differentiation of F0-F1 from F2-F4 in their retrospective pediatric study. They compared SWE measures (using the same US Canon Medical System) with percutaneous biopsies performed within 6 months (11). In spite of a relatively strong correlation between SWE measures and grade of fibrosis (12,19), staging liver fibrosis with SWE remains a challenge due to the considerable overlap between various grades of fibrosis (8,10).

In a similar way, Garcovich et al reported SWE measures concordant with the current study. They investigated pediatric NASH patients and found an AuROC of 0.92 (95% CI: 0.86-0.98), with an optimal cutoff of 5.1 kPa (sensitivity, 85%; specificity, 95%) to differentiate any grade of fibrosis from absence of fibrosis (19). Several other studies, comparing biopsy with SWE values in pediatric cohorts, report cutoff values for normal liver tissue, that is, nonsignificant fibrosis, slightly higher as compared to the current study (between 1.56 and 1.67m/s = 7.0 and 8.5 kPA) (12,13,15).

Choosing an approach with cutoff values for significant fibrosis will of course generate higher cutoff values. In the study by Alhasmi et al, a cutoff value for significant liver fibrosis of >1.89 m/s (approximately 11 kPa) was recommended, yielding a sensitivity of 74%, respectively, a specificity of 78%. Similarly, Phelps et al, using pSWE, recommended a cutoff ≥2 m/s for those who are likely to have significant fibrosis (15).

Regardless if cutoff values for significant fibrosis or nonsignificant fibrosis is reported, large differences in recommended cutoff values exists. Tutar et al, also compared biopsy with SWE values in children, reporting much higher SWE values for all grades of fibrosis and suggested a cutoff value 10.4 kPa to separate F0-1 from higher grade of fibrosis (16). Similary, Franchi-Abella et al reported high cutoff (7.9 kPa) for differentiating F0 from F1 or higher grades (8) and Dhyany et al suggested 8.8 kPa to differentiate significant fibrosis (≥F2) from lesser degrees of fibrosis (F0-1) (8,17). Differences between these studies and the current study are likely due to methodological issues and technical advances in SWE in recent years. For example, neither of those studies report reliability measures or used IQR/median ratio as a quality indicator (9). Studies not adhering to updated recommendations should be interpreted with caution.

Patients with suspected liver disease (i.e., elevated serological markers) with F0-1 have higher SWE measures compared with healthy controls (5). In healthy pediatric livers, using the 2D-SWE technique and updated recommendations for SWE procedure, a median SWE value between 4.3 and 5.0 kPa (with different manufacturers) has been reported (4,10). Anesthesia has been reported to generate higher SWE values. Taken this into consideration, our suggested cutoff of 4.5 kPa lies well within the range for normal values in children. Altogether, it is highly unlikely that if obtaining values below this cutoff that the child has significant fibrosis.

No correlation was found between SWE values and hepatic serological markers, which is in concordance with the few existing studies investigating this relation (2). Also, a weak correlation between SWE measures and inflammation was confirmed (2,5,6), indicating that other measures are needed to evaluate liver inflammation noninvasively.

Shear wave dispersion (SWD) is a newly developed imaging technique that use the dispersion slope of the shear waves to reflect tissue viscosity (21). A preclinical study report that SWD could be a better predictor of inflammation than the speed of the shear waves. Further studies are encouraged to investigate if the combination of SWE and SWD further can improve the diagnostic accuracy of using elastography as a noninvasive diagnostic tool regarding liver disease.

There is growing evidence that longitudinal change in LS can aid in diagnostic workup (9,10) and that combining the evaluation with certain serological markers, for example, aspartate transaminase/alanine aminotransferase ratio (23), can be used to sharpen noninvasive diagnostics further. Including a wide variety of liver diseases, as in the current study, is a strength but of course limits conclusion regarding diagnostic performance of SWE in relation to specific diseases. Any such subanalysis, stratified for disease, was however not the aim of this study. It remains to evaluate if the assessment of liver disease in children with SWE could be improved in diagnostic performance by complementary information from the conventional ultrasound examination, laboratory findings, stratification of disease and an individual long-term follow-up of SWE values.

## LIMITATIONS

In spite of a fairly large cohort, the subgroups in some grades of fibrosis were small, which of course is a limitation. General anesthesia has in few studies been reported to generate significantly higher SWE measurements why our reported cutoff values cannot be directly transferred to a nonsedated situation (2,6,24). We have an ongoing study that aim to evaluate the difference in SWE values between anesthesia and without, using updated recommendations. In that study, we will also include a control cohort. Sampling error exist both with SWE measurements as well as with liver biopsy. The grade of fibrosis was possibly overestimated in a few cases in the current study, since a higher scoring was always chosen even if the specimen only included a small sample of the higher grade. Conversely, the strive for an acceptable <30 IQR/median ratio might in fact result in sampling error in terms of underestimating the SWE measurements, obtaining measurements in the most homogenous parenchyma (6,25). This might have influenced the results, especially in conditions with heterogeneous parenchyma, that is, advanced fibrosis or in congestive hepatopathy (25). In addition, a subcostal approach, which was necessary in some patients with split transplants, might have introduced higher SWE values, as this approach is suboptimal as compared to the recommended intercostal approach. However, care was taken not to induce pressure with the transducer when performing subcostal measurements.

The Metavir and Ishak scoring systems for fibrosis is commonly used is other studies, Batts & Ludwig scoring system was used in this study since this scoring is applied clinically at the study site. However, other studies have shown correlation between SWE and histology irrespective of scoring systems (5).

## CONCLUSIONS

2D-SWE ultrasound, with free breathing, can distinguish no/mild fibrosis from moderate/severe fibrosis in children with suspected/established liver disease with good diagnostic accuracy. Our results show that in pediatric patients when the indication for biopsy is to rule out significant fibrosis, SWE can be considered an alternative. It remains to evaluate if the diagnostic performance can be further improved by considering the pooled results from the ultrasound exam with SWE, laboratory findings, and the individual long time follow up.

## ACKNOWLEDGMENTS

We thank and acknowledge Dr J. Ahlin, Dr K. Mack, Dr M. Brink, Dr L. Steen, Mrs A. Balchman, Mrs E. Lundberg, Mrs P. Björkman and Mrs A. Bjarnehed for their assistance in patient enrollment, data sampling, analysis, and support in image processing.

## Supplementary Material


