# Supplementary material for: Shear Wave Elastography in the Differentiation of Nonfibrotic Versus Fibrotic Liver Disease in Children: A Prospective Study With Histological Correlation
Source: JPGN Rep. 2021 Dec 10;3(1):e156. doi: 10.1097/PG9.0000000000000156 (PMC10158420; doi:10.1097/PG9.0000000000000156)
Supplement: Supplementary file 1 [file pg9-3-e156-s001.pdf]

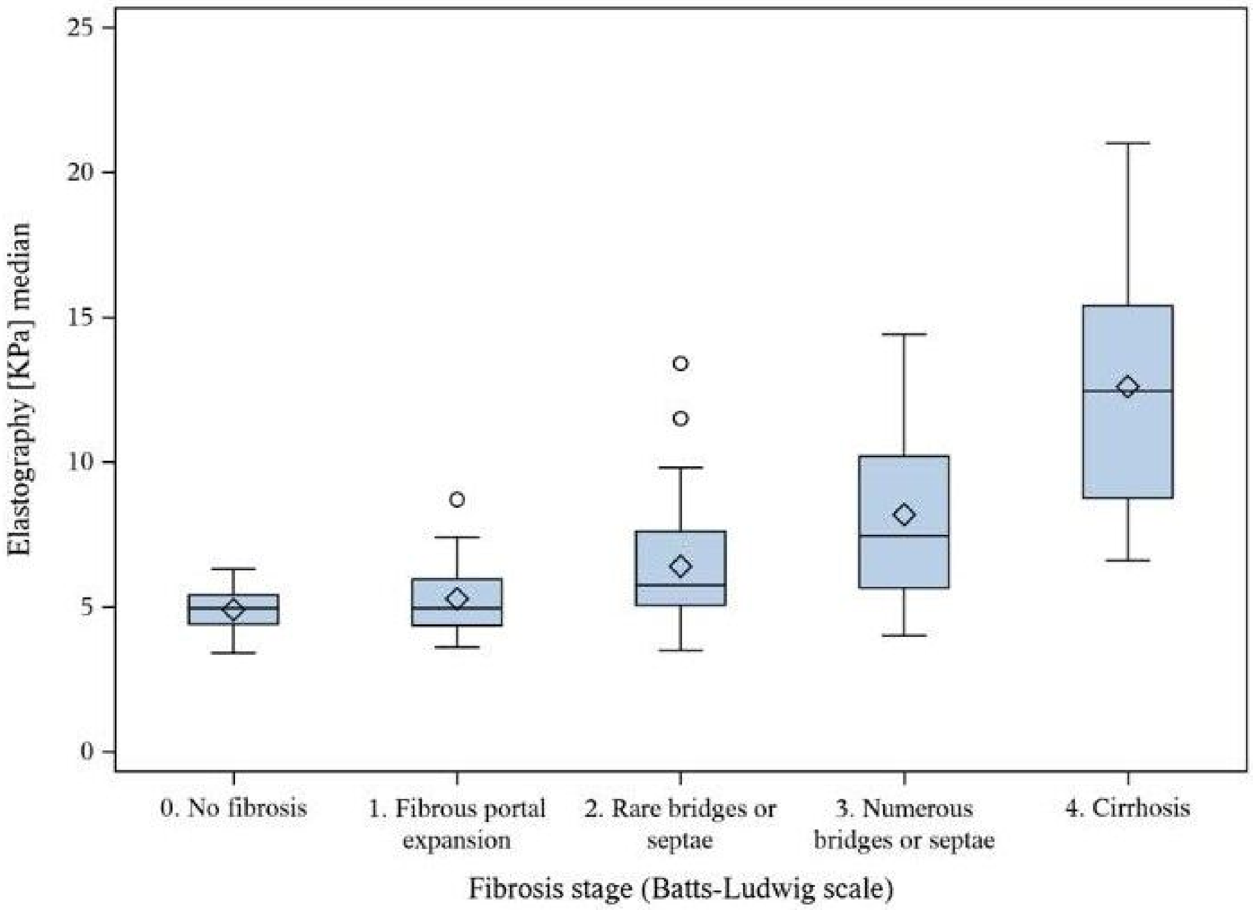

25
20
15
10
5
0
Elastography [KPa] median
0. No fibrosis
1. Fibrous portal expansion
2. Rare bridges or septae
3. Numerous bridges or septae
4. Cirrhosis
Fibrosis stage (Batts-Ludwig scale)

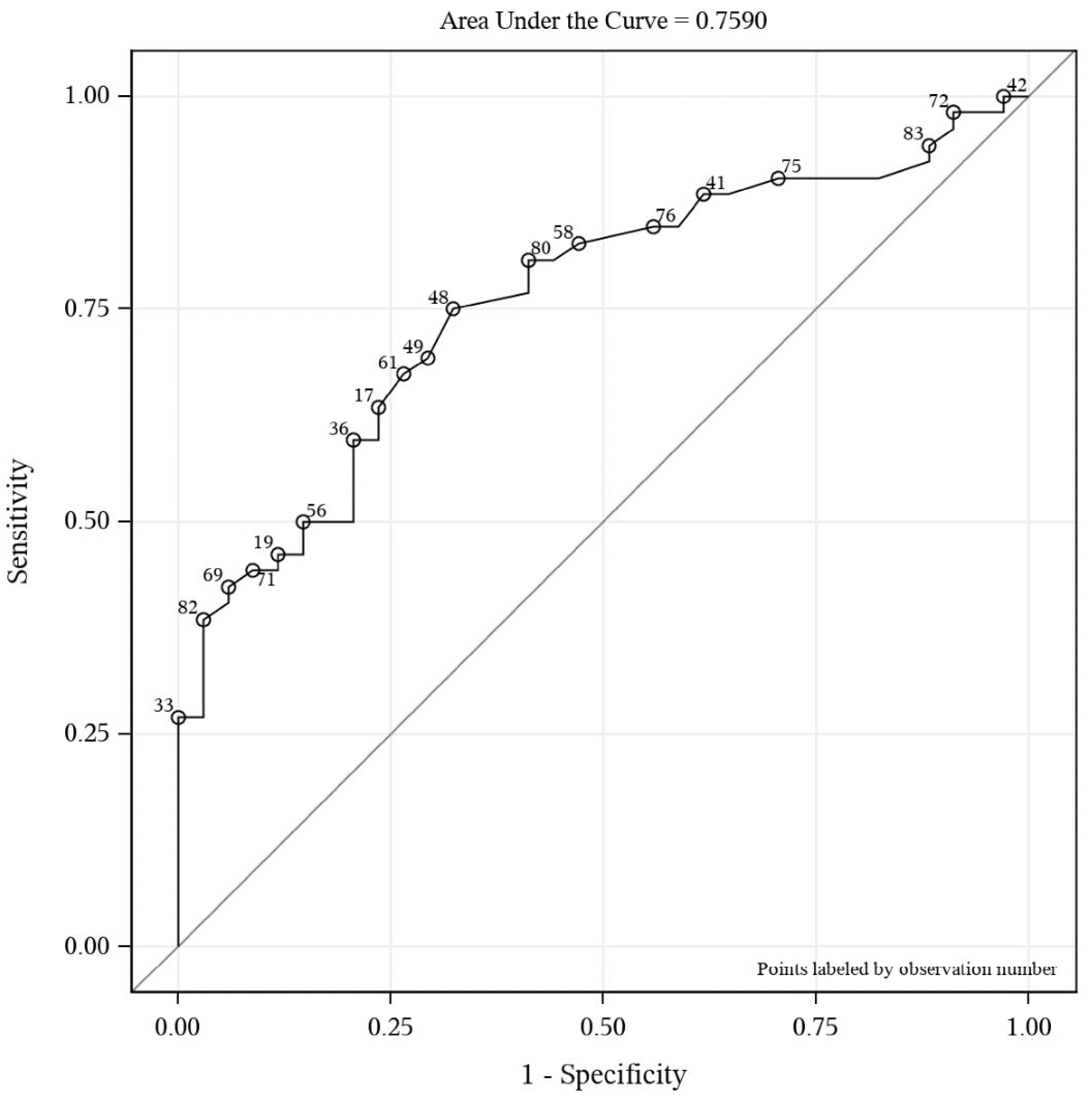
Area Under the Curve = 0.7590
Sensitivity
1 - Specificity
0.00
0.25
0.50
0.75
1.00
Points labeled by observation number
33
82
69
71
19
56
36
17
61
49
48
80
58
76
41
75
83
72
42

**Table, Supplemental Digital Content 1; SIndication for liver biopsy**

| Indication for biopsy/clinical diagnosis | Number of individuals (biopsies) |
|---|---|
| Portal Hypertension | 3 |
| Autoimmune hepatitis | 11 (12) |
| Livertransplants (of which 3 acute biopsies; 4 biopsies displayed some kind of rejection) | 24 (25) |
| Unspecified - increased serological livermarkers | 14 |
| Cystic Fibrosis | 3 |
| Primary and autoimmune sclerosing cholangitis | 9 |
| Non-alcoholic steatohepatitis (NASH), | 2 |
| Hepatitis B | 5 |
| Unspecified cholestatic disease | 4 |
| Intestinal failure-associated liver disease (IFALD) | 1 |
| Neonatal Cholestasis | 1 |
| Congenital disorder of glycosylation | 1 |
| Wilsons Disease | 3 |
| Heart transplant w liver disease | 1 |
| Nieman Pick Type 1 | 1 |
| alfa 1 antitrypsin deficency | 1 |
